# Barriers and Facilitators of Use of Hydroxyurea among Children with Sickle Cell Disease: Experiences of Stakeholders in Tanzania

**DOI:** 10.3390/hemato2040048

**Published:** 2021-11-28

**Authors:** Manase Kilonzi, Hamu J Mlyuka, Fatuma Felix Felician, Dorkasi L Mwakawanga, Lulu Chirande, David T Myemba, Godfrey Sambayi, Ritah F Mutagonda, Wigilya P Mikomangwa, Joyce Ndunguru, Agnes Jonathan, Paschal Ruggajo, Irene Kida Minja, Emmanuel Balandya, Julie Makani, Nathanael Sirili

**Affiliations:** 1 School of Pharmacy, The Muhimbili University of Health and Allied Sciences, Dar es Salaam P.O. Box 65013, Tanzania;; 2 School of Nursing, The Muhimbili University of Health and Allied Sciences, Dar es Salaam P.O. Box 65013, Tanzania;; 3 School of Medicine, The Muhimbili University of Health and Allied Sciences, Dar es Salaam P.O. Box 65001, Tanzania;; 4 School of Dentistry, The Muhimbili University of Health and Allied Sciences, Dar es Salaam P.O. Box 65013, Tanzania;; 5 Department of Development Studies, School of Public Health and Social Sciences, Muhimbili University of Health and Allied Sciences, Dar es Salaam P.O. Box 65013, Tanzania;

**Keywords:** hydroxyurea, barriers, facilitators, utilization

## Abstract

Factors contributing to low use of HU among SCD patients exist in high-income countries. The latter leaves a drift of literature on factors for low utilization of HU in developing countries. This study aimed to explore the factors influencing the use of HU in the management of SCD in Tanzania. A qualitative study was employed to interview purposively selected participants for this study. The in-depth interviews were conducted with 11 parents of children with SCD, four medical doctors working at sickle cell clinics, and two representatives of the national health insurance fund (NHIF). Interviews were audio-recorded, transcribed, and thematically analysed. Barriers identified were misconception of parents on SCD, financial constraints, regulatory restrictions, worries and fears of medical doctors on the acceptability of HU, shortages of laboratory equipment and consumables, and limited availability of HU. Adequate knowledge of the parents and medical doctors on SCD and HU and opportunities for HU accessibility were the facilitators identified. The utilization of HU by the individual with SCD is affected by several factors, from individual to policy level. Nevertheless, parents of children with SCD and medical doctors working in sickle cell clinics demonstrated good knowledge of the diseases and HU.

## 1. Introduction

SCD is a haematological genetic disorder that is characterized by several symptoms, including haemolysis, obstruction of blood vessels by sickled red blood cells, tissue hypoxia, and other clinical complications [1]. While SCD causes morbidity and can result in mortality, it is also associated with economic burden for patients, their families, the health care systems, and thus the whole nation. Worldwide it is estimated that, between 2010 and 2050, about 14 million newborns will be born with SCD, of which 82% will be from Sub-Saharan Africa [2]. Tanzania is among the Sub-Saharan African (SSA) countries with a high burden of children born with SCD. Worldwide, Tanzania is among five countries with the highest number of newborns with SCD (11,000 SCD births annually) [3]. Furthermore, a study performed by Makani et al. (2011) reported that 13% of Tanzanians are sickle cell carriers [4].

Several interventions are currently used to relieve symptoms and complications of patients with SCD. HU induces the production of foetal haemoglobin (HbF) in both children and adults with SCD [1], hence minimizing the effects of HbS [5]. Studies reported that the benefit of HU to patients with SCD outweighs its risks. Among the observed benefit of HU to patients with SCD are decreased pain crisis, reduced need for blood transfusions, decreased hospital stays, less damage to organs, reduced costs of patient care, and reduction in early mortality [6]. Other interventions include early diagnosis through newborn screening (NBS) programs, infection prevention by vaccination and antibiotic prophylaxis, malaria prevention, timely diagnosis and management of complications, and bone marrow transplantation. The establishment of sickle cell clinics in different health facilities in Tanzania is a key strategy implemented to increase coverage of healthcare services targeting patients with SCD.

In Tanzania, patients with SCD are routinely given folic acid (5 mg/day), daily oral penicillin to prevent pneumococcal infection, healthy education, especially on malaria prevention by minimizing mosquito bites (emphasis on using insecticide-treated bed nets), and early healthcare-seeking behaviour. Blood transfusion is indicated for patients with haemoglobin <5 g/dl or heart failure [3]. Besides, some patients already use HU. A study conducted in Tanzania reported that patients with SCD using HU had increased foetal haemoglobin (HBF) and had a positive correlation with clinical outcomes [7]. Unfortunately, despite the benefit of HU, the literature reports that this medicine is underutilized in several SCD endemic regions, including in developed countries [8]. The situation is worse in SSA, regardless of its 75% contribution to the global burden of SCD. For instance, HU utilization in Tanzania was found to be 1% (10 people out of 1000) [7]. Anecdotally, lack of knowledge on the benefit of HU among health care providers, poor accessibility of the medication, fear of toxicity, and lack of health insurance could be reasons for the underutilization of HU.

A study conducted in the United States of America (USA) on practice patterns and barriers in the utilization of HU in children reported the following challenges: poverty, fear of side effects, laboratory monitoring process, fear of taking medication, and doubt of HU efficacy [9]. Obtaining a refill from the pharmacy and coming for follow-up have been reported as patient-related barriers. Furthermore, a study performed by Jose et al. (2019) reported that patients’ misperception about HU side effects, poor knowledge on the benefit of HU, and poor health literacy could be the reason for the observed HU underutilization [10].

While different factors influencing HU utilization among patients with SCD are documented in higher-income countries, less is documented in low- and middle-income countries. Taking into account the benefits of using HU for patients with SCD, it is important to uncover factors that influence the utilization of HU in Tanzania. This study aimed to explore the factors influencing the use of HU in the management of SCD in Tanzania.

## 2. Materials and Methods

### 2.1. Study Design and Setting

An exploratory qualitative study was employed to interview seventeen purposive selected participants for this study between November 2020 and January 2021. The in-depth interviews (IDIs) were conducted with 11 parents of children with SCD, 4 medical doctors working at sickle cell clinics from 4 public hospitals: Muhimbili National hospital, Temeke, Amana, and Mwananyamala Regional Referral Hospitals (RRHs), and 2 representatives of the national health insurance fund (NHIF). The four sickle cell clinics were selected because they are within public hospitals and have been operating for at least 2 years at the time of this study, and the IDIs with medical doctors were conducted in the respective health facility. IDIs with the parents were conducted at Muhimbili University of Health and Allied Sciences (MUHAS). IDIs with the NHIF representatives were conducted at the headquarters office in Dodoma, Tanzania.

### 2.2. Participants and Recruitment

A purposive sampling strategy was used to recruit medical doctors from the selected public hospitals with sickle cell clinics coordinated by the MUHAS–sickle cell program in Tanzania. A similar sampling strategy was used to recruit parents of children with sickle cell disease from the MUHAS–sickle cell program database (patient registry). The characteristics of these children included those either on HU or have stopped but used the medication for at least three years, children with health insurance, and others with no health insurance. The representative from the NHIF department was purposely selected based on their working experience with the insurance fund.

We conducted IDIs with 11 parents of children with SCD, out of which 8 were females and 3 males. The median age of the parents was 42 years with a range of (24–52) years. Except for one parent, the rest were married, and the majority had attained primary or secondary education and were self-employed. Two parents had children who were not covered by NHIF. Furthermore, we conducted IDIs with 6 medical doctors, of which 4 were working in sickle cell clinics and 2 at the NHIF, and 3 were males. The experience of medical doctors was above 5 years for those working at NHIF and an average of 2 years for those working at the sickle cell clinics in the visited health facilities.

### 2.3. Data Collection Procedures

The IDIs were conducted between November 2020 and January 2021. We collected data using a semi-structured interview guide in the Kiswahili language, the native language for both participants and the researchers. The questions were constructed based on the existing literature and experiences of the researchers on the use of HU in managing sickle cell disease. Different interview guides were used for parents, medical doctors, and NHIF representatives to explore factors influencing the use of HU in the country (Table 1). The interviews were conducted by the first (MK) and second (HJM) authors. Because some children attend SCD clinics at Dar es Salaam city but live far away from the city, we conducted 6 IDIs physically and 5 IDIs via phone calls. To ensure the comfortability and availability of the participants, we contacted them one day before the day of the interview. Before each interview, the researcher requested informed consent for each participant (written physical interviews and oral via phone interviews). This was performed by explaining to each participant the purpose of the study and how the information will be collected, benefits, and their rights to withdraw, as well as confidentiality. Informed consent included consent for the audio recording of the interview.

### 2.4. In-Depth Interviews

Seventeen (17) in-depth interviews were conducted using a semi-structured interview guide to explore the facilitators and barriers towards the use of HU in managing patients with SCD. The interviews were conducted in designated rooms chosen to ensure participant privacy and comfortability. The semi-structured interview guide included open-ended questions and probes to explore and understand better all issues of relevancy to the use of HU. The questions asked evaluated participants’ knowledge and perception on SCD and HU, availability, affordability, and acceptability of the HU, availability of the compulsory laboratory tests, policy-related challenges, and their recommendations. The interview guides were modified twice following the first and the fifth interviews with the parents. Participants responded to all questions during the interviews. All interviews were audio-recorded to capture the information provided by the participants. To supplement the audio recorded information, notes were taken on the key issues that emerged during the interviews including non-verbal information for the physical interviews. We stopped at the 17th interview as there was no new information that was coming; we therefore considered this as our point of data saturation. Each interview lasted between 25 to 30 min.

### 2.5. Data Analysis

The audio-recorded data were transcribed verbatim. The transcripts and the field notes were read and re-read to familiarise with the content and context before the actual analysis process. We used the thematic analysis approach to analyse the collected information. The analysis started by developing the initial codebook based on the study objectives, field notes, and interview guides. The codebook was refined from themes that emerged during the analysis. Data analysis was conducted by six researchers, namely M.K., H.J.M., D.L.M., G.T.M., G.S., and W.P.M., who were working in pairs. The coding was performed with two groups where the first and second authors led the groups. To ensure reliability, the first two transcripts were coded by each group separately, and then they compared the codes for agreement on the final codes and coding. The generated codes were grouped into the respective pre-determined code through comparisons. Then, the frequency of appearance of the related codes generated a sub-theme and finally, themes were developed following the contextualization and conceptualization process. The process of sub-theme and theme generation involved all authors.

## 3. Results

The findings are classified into two major themes that include barriers and facilitators to the use of HU among SCD patients. The barriers include individual barriers (parents’ myth and misconception regarding SCD, medical doctors’ worries and fears to prescribe HU), health facility barriers (lack of infrastructural support, and limited availability of HU in health facilities), and health system barriers (limitation of HU insurance coverage, high cost of HU and restrictive policy on the use of HU). Facilitators regarding the use of HU include having adequate knowledge about SCD and the use of HU in its management (parents’/guardians’ awareness on the disease and the use of HU and medical doctors’ knowledge about SCD and the use of HU in its management) and opportunities for HU accessibility to patients with SCD (readiness of health insurance to cover or to offer the special permits and availability of HU in health facilities). Table 2.

### 3.1. Barriers towards the Use of HU in Managing Patients with SCD

The analysis of barriers towards utilization of HU among patients with SCD generated three themes that include individual barriers, health facility barriers, and health system barriers.

### 3.2. Individual Barriers towards Utilization of HU

Two sub-themes emerged from this theme that is parent’s myths and misconceptions regarding sickle cell disease and the worries and fears of the medical doctors in prescribing HU for SCD patients.

#### 3.2.1. Parent’s Myth and Misconceptions Regarding SCD

Parents/guardians reported that getting SCD is God’s plan and that it can be curable. Other parents believed that patients with SCD patients do not live long, they usually die within a short period, and that there is no need to invest a lot or even to treat them. Other parents went further and said that SCD is mainly attacking the poorer, and they had a strong belief that rich people do not get this rare disease. One participant said:

“……… me and her mother we did some tests for sickle cell and were found negative ………… so I think this is God plan because if it is an inheritable disease even in our family we should have an individual with SCD disease, but in our family, no one has SCD disease”

(Patient 5).

#### 3.2.2. Medical Doctors’ Worries and Fears to Prescribe HU

Medical doctors reported having worries and fears about prescribing HU for SCD patients because they think that parents/guardians would blame them in case of any adverse effects of the drug. They also reported that their worry is on the parent’s awareness of the side effects of the drug because they can access information of HU in different reading sources including google. Conversely, they said that some parents are not aware of the drug, which may affect the adherence even if they will decide to prescribe for SCD patients. The cost introduced fear to some physicians that not all patients would afford the cost of HU and hence affect its adherence. One of the medical doctors said:

“The issue is most of our patients have information regarding the medication before reaching the clinic, and sometimes the information is wrong. So, some patients/parents are afraid of using hydroxyurea because they know that is used by cancer patient ……………. Some think that the medication will affect their reproductive organ and they will not be able to reproduce”

(Medical doctor 3).

### 3.3. Health Facility Barriers towards the Use of HU

#### 3.3.1. Lack of Infrastructural Support

Medical doctors said a shortage of laboratory equipment and consumables in their facilities limit the prescription of HU to some patients. Medical doctors responded that it is important to conduct some preliminary tests such as reticulocyte count and haemoglobin level before initiating HU to an individual. The tests are also required during follow-up visits before HU refill but are limited in most of the health facilities. In addition, one medical doctor said that the absence of paediatric formulation poses a challenge during the prescription of HU to the paediatric population. Nevertheless, some medical doctors think that only specialists, particularly haematologists, are the ones allowed to prescribe HU.

“The challenge on hydroxyurea is on dose especially for paediatrics because the capsule has 500 mg, so to get a dose for paediatrics is difficult. For example, a child is supposed to receive 300 mg, it is difficult to divide the capsule”

(Medical doctor 3).

#### 3.3.2. Limited Availability of HU in Health Facilities

Parents reported facing difficulties in accessing the medicine because few community drug outlets sell HU and that it is available only in tertiary level hospitals. Medical doctors reported referring patients to tertiary hospitals only to access the HU. One of the medical doctors said that, due to some restriction of NHIF or sometimes insufficient availability of HU in hospitals, patients receive few pills, which in turn increases their hospital visits for refilling.

“… currently HU is available in the tertiary hospital, for regional referral hospitals they are not yet. Therefore, if we encounter a patient who needs HU and has all the criteria for using HU, we referred the patient to a tertiary hospital to get the medication and our responsibility is to monitor his/her progress

(Medical doctor 3).

### 3.4. Health System Barriers

#### 3.4.1. Limitations of HU Insurance Coverage

Parents whose children were covered by health insurance said that they face difficulties in accessing HU using the NHIF card. The challenge faced by the parents was the requirement of having a special permit from the NHIF before issuing HU. Public health facilities are missing the NHIF office to facilitate issuing the permits. Therefore, parents were forced to leave the hospital to find the NHIF office for approval to then return to the facility to get the prescription of HU. This bureaucracy of NHIF forces the parents to either buy HU in nearby community drug outlets or return home without the medication.

“Insurance services have a lot of bureaucracy, especially issue of a special permit. Patients with insurance have a lot of offices to attend before getting the services”

(Parent 3).

One of the medical doctors reported that although NHIF covers HU in its package, patients still need a special permit to obtain the drug. These restrictions in provisional HU by NHIF prolong patient waiting and hospital stay time that in turn increases the cost of treatment and later causes dose skipping and poor adherence of HU by the patients. Another participant said that some HIFs allow their beneficiaries to access HU after 1 year of joining the agency, which is different from the NHIF that is being used by the majority.

“….. I think NHIF need to remove some restriction to enhance the availability of hydroxyurea, because the drug is of high importance. For example, a patient from upcountry traveling to a distance tertiary hospital to find hydroxyurea but because of restriction he/she will receive only one-month capsules. This makes patients travel every month to get hydroxyurea, which is not easy for our people”

(Medical doctor 4).

#### 3.4.2. High Cost of HU

Parents reported that HU is sold at a high price that ranges from 1000 to 3000 Tanzanian shillings for one capsule of 500 mg or TZS 2000 to 6000 for two capsules. Since patients are supposed to take the drug every day, parents said that one of the reasons for poor compliance to HU is a financial constraint that makes them unable to buy medicine every time.

“…. she started using hydroxyurea before getting stroke but the medicine was too expensive, so I failed to afford to purchase hydroxyurea every day therefore she stopped. Then I managed to get health insurance for her, but unfortunately, she has already suffered from stroke”

(Parent 6).

A medical doctor also stated that because of the high price of HU, most of the parents cannot afford to purchase monthly doses for their children, and this is the major reason for poor adherence and compliance. Another medical doctor said that initiation and monitoring of HU must involve the conduct of some laboratory tests; however, because of financial challenges, the majority of their patients cannot afford the cost and hence do not perform the tests.

Sometimes issues related to the economy, you find an individual with SCD in need of HU but because of poverty he/she cannot afford to get HU. Therefore, even if you prescribe HU, it is obvious that the patient will not afford the cost of HU

(Medical doctor 1).

#### 3.4.3. Restrictive Policy on the Use of HU

An NHIF representative reported that, according to the Tanzanian policy on medication, all anticancer should be available in the health facilities that are authorized to offer chemotherapy services. Therefore, HU being anticancer limits its availability in many health facilities that do not administer chemotherapies for cancer patients. This restrictive policy was reported to be a barrier for HU to be used in managing patients with SCD.

“Unfortunately, HU is classified as anticancer, so all anticancer such as methotrexate although we know are used to manage non-cancers illness (……) under the national health insurance fund, those types of medication require a special permit”

(NHIF representative 2).

### 3.5. Facilitators towards the Use of HU

The analysis of facilitators of the utilization of HU by patients of SCD generated two themes that include having adequate knowledge of SCD and HU use in its management and opportunities for HU accessibility to patients with SCD.

### 3.6. Having Adequate Knowledge on SCD and HU Use in Its Management

Parents/guardians and medical doctors’ knowledge on SCD and the use of HU in managing these patients were seen to influence the utilization of HU.

#### 3.6.1. Parent’s Awareness of the Disease and the Use of HU

Parents reported being aware that SCD is an inheritable disease and that there is a possibility of the couple delivering kids with or without SCD. One of the parents went further and talk about sickle cell anaemia (Hb (SS)) and sickle cell trait (Hb (AS)). Parents further said pre-marital screening is the option to prevent birthing children with SCD. Despite the demonstrated knowledge, few parents, especially men, still believed that men do not carry the sickle cell gene.

“(…) before marriage, partners should undergo premarital screening to know their status with regards to SCD, it will help in preventing children born with SCD”

(Parent 7).

Parents were able to explain the complications of SCD. The majority said that SCD is associated with anaemia, jaundice, fever, and severe pain. Besides, swelling of the fingers/toes, paralysis of the legs, hand, and mouth, stroke, joint pain, and prolong hospital stay were mentioned as complications of SCD. One parent claimed that her child usually has abdominal swelling and constipation. Parents are aware that, in the management of SCD, several tests are usually performed, and few tests were mentioned during the discussion, including full blood picture, white blood cell count, kidney function test, and urine and stool analysis. When asked about the medications frequently used by their children, the following were mentioned; folic acid, chloroquine, penicillin V, HU, carbamazepine, vitamin D, blood transfusion, fluid replacement, and pain killers such as paracetamol, diclofenac, and ibuprofen.

“After being diagnosed with SCD, she started using folic acid and penicillin V (….) followed by malaria pills, chloroquine as malaria chemoprophylaxis (.…….) she suffered a stroke when reached 6 years and the medical doctor said she needs to start using hydroxyurea”

(Parent 11).

Parents whose children use HU said that the medication alleviates pain and is helpful. They went further to say that HU reduces frequent hospitalization, and blood transfusion reduces morbidity, raises body immunity, minimizes chances for stroke, and reduces frequent vaso-occlusive pain crisis and stroke. Conversely, parents said that HU, similar to other medications, has few side effects which are tolerable, including general body malaise, constipation, and tremors. Other parents said that thus far, they did not observe any side effects due to HU, and one parent responded that HU has no side effects.

“To be honest hydroxyurea has no any side effect so far, my child is going to school as a normal child”

(Parent 4).

#### 3.6.2. Medical Doctors’ Knowledge about SCD and the Use of HU in Its Management

Medical doctors described SCD as a genetic disorder inherited from parents. SCD affects red blood cells in which their shape resembles the moon. One medical doctor added that the moon-like shape is the reason for the pain crisis faced by SCD patients. Medical doctors said that because SCD affects blood level, it eventually damages multi-organs, and the red blood cells are extensively destroyed, leading to anaemia and yellowing (jaundice). In addition, joint pain, swelling, and growth retardation are also complications of SCD. Medical doctors expressed that, because of several complications, SCD patients use several medications such as folic acid, antibiotics, penicillin V and chloroquine as prophylaxis of pneumonia and malaria, and pain killers include paracetamol, ibuprofen, and HU.

“…… SCD is an inheritable disease, in which a patient inherits red blood cell mutated genes from both father and mother. The disease affects red blood cells which result in changes in the shape of the cell”

(Medical doctor 4).

Regarding HU, one medical doctor said that it is an anticancer with the ability to induce foetal haemoglobin. Most of the medical doctors demonstrated a positive perception of the benefits of HU when used in the management of SCD. Some said that HU is highly effective with good growth outcomes, HU reduces the frequency of pain crisis, and HU relieves complications and increases haemoglobin level. In addition, medical doctors voiced that a patient on HU is at risk of the following side effects: thrombocytopenia, reticulocyte reduction, reduced HB level, exposing patients to infections, and causing skin problems, headache, myelosuppression, anaemia, and leukopenia. Medical doctors said that to minimize the risk of the side-effect, they usually provide a one-month dose of HU during initiation and when patients return, they perform examinations coupled with laboratory tests before prescribing the next dose. If the patient presents with no complications from HU after three months, they start prescribing 3-month doses. Furthermore, one prescriber insisted that HU is contraindicated during pregnancy.

“(……) the majority when using HU are progressing well, those who were getting attack frequently usually are reduced with HU. For example, if an individual with SCD was getting attacks 2 times per month, with HU may survive 3 months free of attack. Therefore, it helps”

(Medical doctor 2).

### 3.7. Opportunities for HU Accessibility to Patients with SCD

Two sub-themes emerged from this theme, namely readiness of health insurances to cover or to offer special permits for HU and availability of HU in health facilities.

#### 3.7.1. Readiness of Health Insurance to Cover or to Offer Special Permits for HU

Medical doctors said that some patients access HU through NHIF. They further reported that NHIF reduces the cost of treatment because patients can access both laboratory tests and medication. Medical doctors said that, through NHIF, patients adhere well to the medication. One medical doctor suggested that if we want to increase the utilization of HU, we must convince more parents to find health insurance for their children.

“(……) in general health insurance helps, help a lot in accessing treatment and we usually encourage patients to have health insurance. Currently, the most available health insurance is NHIF

(Medical doctor 1).

#### 3.7.2. Availability of HU in Health Facilities

Both parents and medical doctors confirmed that HU is available in some community pharmacies and that there is no restriction in accessing the medication. In addition, HU is available in private hospitals of all levels. One parent witnessed that she had to shift her children from a public to a private hospital sickle cell clinic to access HU easily.

“(………). when she started using hydroxyurea (……) I was purchasing the medication through a community pharmacy after being prescribed by a doctor. The doctor insisted that due to the condition of your child, she needs hydroxyurea, can you purchase the medication? Because, the health of my child is my number one priority, I was buying hydroxyurea from the community pharmacy until I managed to secure health insurance for her

(Parent 4).

## 4. Discussion

Our study explored the barriers and facilitators towards the utilization of HU among patients with SCD in Tanzania. We managed to obtain parents’, medical doctors’, and NHIF representatives’ experience on the barriers and facilitators for use of HU among children with SCD. The study managed to identify three barriers related to individuals, health facilities, and the health system. The study also identified two facilitators, which if well utilized, will increase the utilization of HU among SCD individuals; these are knowledge of parents and medical doctors on SCD and uses of HU and opportunities for HU access to patients with SCD.

The study found that parents’ myths and misconceptions about SCD affect the management of their children. Parents believe that individuals with SCD usually die early and do not need to spend more energy to treat them while others perceive SCD as God’s will. Myths and misconceptions on health services have been reported to affect several health programs, including uptake of medicines for mass drug administration against schistosomiasis, lymphatic filariasis infection[11], and vaccines. For instance, currently, the world is facing the COVID-19 pandemic, and despite the availability of the vaccines, individuals worldwide are hesitating to be vaccinated because of the existing myths about vaccines [12]. To enhance the utilization of HU among individuals with SCD, parents’ and patients’ awareness of the disease should be addressed. This has been explained by Musuka et al. (2018) on a tuberculosis study in which the success of the directly observed treatment (DOT) depended on the prevailing perceptions of the community about the disease, stigma, and awareness of early signs, as well as access to health services [13]. Similarly, the success story behind the uptake of ivermectin in many MDA projects was enhanced by awareness creation [11].

Medical doctors fear that patients will not adhere to HU because of the side effects and because the drug is anticancer. The findings are consistent with what was reported in Nigeria in a study that assessed the level of HU utilization and provider’s related factors in which, among the reported barriers, there were fears of side effects and doubts about its effectiveness [14]. In addition, similar findings were reported in a review conducted in the USA on HU use in SCD individuals regarding the battle with low prescription rates, which reported that providers fear that patients will not use the medication because of side-effects [9]. The literature shows that among the limitations towards medicine compliance by the patients is fear of side effects [9]. It is reported that patients who have used the medicine previously and had side effects, those who witnessed their relatives, or those who read about the medication and became familiar with the side effects are at high chance of not completing the dose. Measures to overcome fears on HU among healthcare providers are recommended and include developing a workable protocol on the use of the drug and pieces of training [15].

Medical doctors said that shortage of laboratory machine and tests in most of the health facilities limit the uses of medicines such as HU, which require preliminary and follow-up tests. One of the medical doctors reported that the laboratory tests are available only in tertiary hospitals. Laboratory tests such as complete blood count, chemistry profile (electrolytes, total protein, albumin level), serum iron, liver function test, and renal function test are recommended before initiation of HU and during follow-up [5]. The laboratory tests are important because uses of HU have been associated with neutropenia, bone marrow suppression, macrocytosis, and elevation of hepatic enzymes [5]. However, in developing countries, because of poverty, the small amount of money budgeted for health is directed towards disease prevention and provision of care, leaving aside other important aspects of health; therefore, the quality of most of the laboratory services is poor [16].

Both parents and medical doctors have said that HU is not readily available, particularly in primary and secondary health facilities. Parents reported that few community pharmacies sell the medication, and one medical doctor said lack of paediatric formulation limits the initiation of HU to most of the SCD children. According to Tanzanian policy, anticancer is allowed to be available in tertiary hospitals only, and currently, HU is classified as anticancer. Our findings are in line with what was reported by Saliba et al. in 2020, in which HU was found to be poorly available in Tanzania [17]. The study reported that out of five community pharmacies visited, three did not have HU, and the major reason for the out-of-stock was low demand [17]. Shortages of medications in health facilities have been reported to cause clinical, economic, and humanistic negative impacts. Similar findings have been reported from the study which was conducted in the Democratic Republic of Congo, whereby poor availability and high-cost limited accessibility of HU existed among SCD patients [18].

Difficulties in accessing HU through NHIF is another barrier mentioned towards the utilization of HU. Medical doctors and parents reported that before obtaining HU a special request should be sent to NHIF for approval. In case the health facility has no office for NHIF, the parent/patient must travel to find a nearby office for approval. These findings are inconsistence with what is reported in a developed country in which lack of a medical home, limited access to comprehensive sickle cell centres, lack of care coordination between comprehensive sickle cell centres and community-based physicians for those children that are geographically isolated from a comprehensive sickle cell centre, and poor transition from paediatric to adult care were reported as health system-related barriers towards utilization of HU [19,20]. In developed countries, health insurance agencies are well established compared to low-middle income countries. The reported disturbance due to the NHIF system could prolong the duration of obtaining HU and poor compliance to the medication. In addition, the movement to the NHIF office requires additional money for transportation, which adds a burden to parents, of which the majority are poor.

Both parents and medical doctors reported that the price of HU is high and that it varies depending on the location of the pharmacy. Parents reported that they purchased the medication from community pharmacies ranging from 1500–3000 Tanzanian shillings (equivalent to USD 0.65–1.3) per 500 mg HU capsule. The price reported is slightly similar to what was reported in 2020 in Tanzania, in which the price of HU was found to be USD 0.27–0.91 [17]. The slight difference may be attributed to the fact that the previous study assessed the price of HU in public hospitals while participants in our study shared experiences from community pharmacies. A systematic review of the implementation of medicine pricing policies in SSA (2021) indicated that the prices of medication are high in SSA [21]. Weak regulatory authorities, corruption, and shortage of local pharmaceutical industries in SSA are the major reasons why the prices of medication are high. For example, in Tanzania, no pharmaceutical industry manufactures HU, despite the feasibility study which has proved the capacity to do so [17]. In addition, there is only one registered market authorization holder (MAH) that imports HU in Tanzania. Nevertheless, the literature shows that individuals with SCD are from a marginal background with low socioeconomic status which poses difficulty in accessing health services and related costs such as transportation [20].

The parents further said that premarital screening can be introduced to reduce the number of children born with SCD. This suggestion is in line with what had been reported in different studies on prenatal screening. A study conducted in Jamaica (2017) on voluntary premarital screening to prevent SCD concluded that future options are a greater role in prenatal diagnosis [22]. Furthermore, a study conducted in Nigeria on the attitude of local government workers towards premarital screening found that 95% of the study objects were positive towards the tests for marital decisions [23].

## 5. Strength and Limitation

Several measures were taken to ensure rigor and trustworthiness i.e., credibility, dependability, transferability, and confirmability of the research findings. These include a purposive sampling of patients, medical doctors in health facilities, and NHIF representatives with experience of more than three years of using HU, two years of attending SCD patients, and more than five years in the insurance scheme, respectively. In addition, in-depth interviews in comfortable and noiseless rooms, as well as the use of Kiswahili language which is spoken by natives, ensured naturalistic, high-quality audio recordings and free-flowing of ideas during the IDI. In addition, the use of a semi-structured interview guide which was constantly evolving allowed flexibility and emergence of ideas. Furthermore, verbatim transcription plus field notes on non-verbal communication reduced context distortion. Finally, the use of thematic analysis and involvement of multiple researchers during data analysis allowed for multifaceted description and interpretation of the findings.

Being a qualitative study, where the researcher is part of the tool for research, it is obvious that some degrees of subjectivity are acknowledged as a limitation. The PI is an experienced practicing pharmacist and a researcher specialized in pharmacology and therapeutics; thus they might have been affected by sympathy and empathy which would have reduced confirmability to this study. However, this was minimized by involving multiple researchers during data analysis. In addition, the study missed some non-verbal communications from five participants who were interviewed online, which might contribute to some degree of context distortion.

## 6. Conclusions

The utilization of HU by an individual with SCD is affected by several factors, including myths about the disease as well as fear of side effects, limited availability, and accessibility of HU. Nevertheless, accessibility of HU in hospitals, community pharmacies and coverage of HU through NHIF enhanced the use of HU by individuals with SCD. The study recommends the need for continuous sensitization or awareness creation on SCD, use of HU, and its safety profile to prescribers and parents. In addition, the responsible authority should institute measures to ensure HU is available and affordable in health facilities without restriction. Furthermore, accessibility of HU through health insurance fund agencies should be smooth to minimize unnecessary disturbances which result in dose skipping and poor adherence.

## Figures and Tables

**Table 1.** Interview guide questions.

S/N	Questions
	Interview guide—Parents
**1**	Thank you very much. I would like to start by a general discussion on your knowledge on the Sickle Cell Disease. Briefly tell me what you understand about Sickle Cell Disease (SCD). (Probe: Meaning of the disease, Causes of the disease (genetical vs a causative agent), Transmission of the disease (congenital vs a causative agent), Treatment of the disease (symptomatic vs full treatment) and Prevention (means of prevention?)
**2**	Thank you for the response. I would like to discuss in brief your medication history. (Probe: Drugs used (Probe for Hydroxyurea, L-glutamine oral powder, Crizanlizumab, Voxelotor, antipain, antibiotics, pneumococcal vaccine, chloroquine, FEFO, BT) and If changing drugs, probe for reasons for the change (S/effects, availability etc))
**3**	Now that we have discussed your drug history, I would like to know if there is any drug you prefer more than the other and why? (Probe: Facility factors such as availability of drug, customer care of providers, providers’ advice, skills, knowledge etc., and Patient related factors for utilization of HU, such as efficacy, side effect, clinic follow-up, knowledge about the drug))
**4**	What do you think the government should do to increase the compliance of Hydroxyurea to SCD patients? (Probe: Incorporate the drug to Insurance, vertical program)
**5**	Is there anything else concerning this topic that you would like to share with me before we close the discussion?
	Interview guide—Medical Doctors
**1**	Could you tell me what drugs are used to manage sickle cell Disease in your facility? (Probe for Hydroxyurea, L-glutamine oral powder, Crizanlizumab, Voxelotor, antipain, antibiotics, pneumococcal vaccine, chloroquine, FEFO, BT)
**2**	You have mentioned Hydroxyurea among others, could you tell me the reasons for you to prescribe/dispense hydroxyurea for SCD? (Probe for facility factors such as availability/affordability of drug, efficacy, side effects, patient compliance etc.)
**3**	What do you think the government should do to increase the compliance of Hydroxyurea to SCD patients? (Probe: Incorporate the drug to Insurance, vertical program)
**4**	Is there anything else concerning this topic that you would like to share with me before we close this discussion?
	Interview guide—National Health Insurance Fund
**1**	Why is HU not covered by health insurances? (Probe: What are the criteria used to allocate a drug under NHIF coverage? What are the criteria used to allocate a drug under vertical program? and What should HU need to meet for it to be covered by NHIF or placed under vertical program)
**2**	Is there anything else concerning this topic that you want to share with me before we close this discussion?

**Table 2.** Summary of findings.

Selected Codes	Sub-Themes	Themes
Getting SCD is God’s will SCD patients do not survive Is a disease of poor people	Parent’s myth and misconceptions regarding SCD	Individual barriers
Patients doubt on HU that it causes cancer and infertility Patients read and know the side effects of HU Patients are not aware of HU uses that affect adherence The cost of HU affect compliance	Medical doctors worries and fears to prescribe HU	
Shortage of lab machine for reticulocyte count Absence of paediatric formulation Lab tests are only available in a tertiary hospital Some facilities are not allowed to have HU	Lack of infrastructural support	Health facility barriers
A special permit is needed to access HU Few pharmacies sell HU Not available in most health facilities Few pills are provided per visit	Limited availability of HU in health facilities	
Permit delay initiation of HU to patient HU is covered by insurance after 1 year of use Some insurance scheme does not cover HU Insurance bureaucracy at initiation Insurance agencies are profit-oriented	Limitations of HU insurance coverage	Health system barriers
HU cost is high Variation in HU price I skip sometimes due to cost	High cost of HU	
HU being recognized as anticancer Restrictions of HU to be used in SCD	Restrictive policy on the use of HU	
The disease is not contagious SS is an inherited disease HU raise blood amount HU use reduces the frequency of hospital visits	Parent’s awareness of the disease and the use of HU	Having adequate knowledge of the SCD
SCD is a genetic disorder SCD is an inherited disease from both parents HU prevent SCD crisis HU reduce morbidity	Medical doctors’ knowledge about SCD and the use of HU in its management	
Insurance help on HU accessibility Insurance reduces the cost of HU Insurance coverage raise HU adherence	Readiness of Health Insurance to cover or to offer special permit for HU	Opportunities for HU access to patients with SCD
Being available in HF without restrictions HU is available in community outlets Availability in private health facilities	Availability of HU in health facilities	

## Data Availability

Data are not available publicly because they contain sensitive interview information and participants did not consent for their interviews to be shared publicly. The data are available from The Directorate of Research and Publication Muhimbili University of Health and Allied Sciences (contact via drp@muhas.ac.tz Tel.: +2552150302–6) for researchers who have criteria to access confidential information.
